# Screening the Expression Changes in MicroRNAs and Their Target Genes in Mature Cementoblasts Stimulated with Cyclic Tensile Stress

**DOI:** 10.3390/ijms17122024

**Published:** 2016-12-07

**Authors:** Liao Wang, Haikun Hu, Ye Cheng, Jianwei Chen, Chongyun Bao, Shujuan Zou, Gang Wu

**Affiliations:** 1 State Key Laboratory of Oral Diseases, West China Hospital of Stomatology, Sichuan University, No. 14, 3rd Section, South Renmin Road, Chengdu 610041, China; wangliaonew@scu.edu.cn (L.W.); dr_chengye@163.com (Y.C.); bulb13@163.com (J.C.); cybao9933@scu.edu.cn (C.B.); 2 China Dental Implantology Center, West China Dental Implantology Hospital, Sichuan University, No. 75 Xiaotianzhu Street, Chengdu 610041, China; helen-8591@163.com; 3 Department of Oral Implantology and Prosthetic Dentistry, Academic Centre for Dentistry Amsterdam (ACTA), VU University Amsterdam and University of Amsterdam, MOVE Research Institute, Gustav Mahlerlaan 3004, 1081LA Amsterdam, The Netherlands

**Keywords:** cementoblast, tensile stress, miRNA, microarray, miR-146b-5p, Smad4

## Abstract

Cementum is a thin layer of cementoblast-produced mineralized tissue covering the root surfaces of teeth. Mechanical forces, which are produced during masticatory activity, play a paramount role in stimulating cementoblastogenesis, which thereby facilitates the maintenance, remodeling and integrity of cementum. However, hitherto, the extent to which a post-transcriptional modulation mechanism is involved in this process has rarely been reported. In this study, a mature murine cementoblast cell line OCCM-30 cells (immortalized osteocalcin positive cementoblasts) was cultured and subjected to cyclic tensile stress (0.5 Hz, 2000 µstrain). We showed that the cyclic tensile stress could not only rearrange the cell alignment, but also influence the proliferation in an S-shaped manner. Furthermore, cyclic tensile stress could significantly promote cementoblastogenesis-related genes, proteins and mineralized nodules. From the miRNA array analyses, we found that 60 and 103 miRNAs were significantly altered 6 and 18 h after the stimulation using cyclic tensile stress, respectively. Based on a literature review and bioinformatics analyses, we found that miR-146b-5p and its target gene Smad4 play an important role in this procedure. The upregulation of miR-146b-5p and downregulation of Smad4 induced by the tensile stress were further confirmed by qRT-PCR. The direct binding of miR-146b-5p to the three prime untranslated region (3′ UTR) of Smad4 was established using a dual-luciferase reporter assay. Taken together, these results suggest an important involvement of miR-146b-5p and its target gene Smad4 in the cementoblastogenesis of mature cementoblasts.

## 1. Introduction

Cementum is a thin layer of mineralized tissue produced by cementoblasts [1,2]. As a unique avascular mineralized tissue, cementum covers the tooth root surface and plays an important role in anchoring teeth through the periodontal ligament and protecting the integrity of root surfaces. The integrity and proper functionality of cementum is a pre-requisite biological basis for masticatory activity and orthodontic tooth movements. Although cementum has a similar biological composition to bone, it exhibits a relatively slower remodeling pattern [3,4]. Its greater resistance to mechanical force than bone is also regarded as the physiological basis for orthodontic tooth movement [5]. During orthodontic intervention, new cementum forms at the tension sites suggesting the stimulating effect of tensile stress on cementum remodeling and regeneration [1,6,7,8,9]. Conversely, in many pathological cases, such as masticatory hypofunction, the compromised mechanical stimuli may cause a significant reduction in the cementum thickness, which can eventually lead to the loss and discontinuity of the cementum [10]. Consequently, mechanical forces play a paramount role in facilitating the maintenance, remodeling and integrity of cementum.

A series of in vitro cellular experiments have corroborated the mechanosensitivity of cementoblasts. Under certain mechanical stress (no matter tensile or compressive), cementoblasts may undergo cementoblastogenesis. Except for a few unique characteristics, such as cementum protein 1 [4], cementoblastogenesis largely resembles the major characteristics of osteoblastogenesis, such as the upregulation of proliferation, bone sialoprotein (BSP) [6,11], osteopontin (OPN) [11] and alkaline phosphatase (ALP) activity [5]. With this principle, osteoinductive growth factors, such as bone morphogenetic proteins (BMPs), have been adopted to regenerate the periodontal complex. Bone morphogenetic proteins (BMPs), which are a group of growth factors within the transforming growth factor-β superfamily, play an important role in osteoblastogenesis and bone regeneration [12]. In osteoblasts, BMP signaling is triggered by binding to serine-threonine kinase receptors (type I and type II) and the subsequent phosphorylation of Smad 1/5/8. The complexes can translocate onto the nucleus with Smad4 and activate the transcription of target gene, Dlx5, which enhances osteogenic differentiation by promoting the transcription of Runx2 and Osterix [13,14]. However, in clinical trials, these therapies have not been as promising as anticipated in regenerating periodontal tissues. Although BMP-2 has been shown to promote dental follicle cells, which are the putative progenitor cells for the periodontium, to differentiate into a cementoblastic/osteoblastic phenotype [15,16], BMP-2, however, inhibits differentiation and mineralization of mature cementoblasts in vitro [17]. These results suggest that the cementoblastogenesis of mature cementoblasts might be differently modulated by mechanical forces compared with osteoblastogenesis. However, the molecular mechanism accounting for the mechanical force-induced cementoblastogenesis remains largely unknown. Hitherto, the extent to which a post-transcriptional modulation mechanism is involved in this process has rarely been reported.

MicroRNAs (miRNAs) are a family of highly conserved small non-coding RNA molecules acting as post-transcriptional suppression factors. miRNAs bind to the specific mRNA recognition sequences that are located in the 3′ untranslated regions (3′ UTRs) of the target mRNA, which leads to mRNA degradation or protein translation interference [18]. Recent studies have demonstrated that miRNAs are differentially expressed between cyclically-stretched and non-stretched periodontal ligament stem cells (PDLSCs) [18,19,20], and the pattern is closely related to the mRNA expression of osteogenic markers, which indicates the important regulatory roles of miRNA in bone remodeling of orthodontic tooth movements. However, hitherto, the miRNA expression pattern of mature cementoblasts has rarely been reported. The understanding of miRNAs and their regulatory networks will provide new insight into the modulating mechanisms in force-induced cementum formation and regeneration.

In this study, we attempted to identify the effects of mechanical forces on cementoblastogenesis and the associated post-translational modulation mechanisms. We adopted a four-point bending apparatus to produce cyclic tensile stress. We examined the effect of different parameters on the cementoblastogenesis in a well-established murine cementoblast cell line OCCM-30 cells (immortalized osteocalcin positive cementoblasts). We screened the miRNA profile and identified potential target mRNAs in the mechanical force-induced cementoblastogenesis.

## 2. Results

### 2.1. The Effect of Tensile Stress on the Cell Morphology and Proliferation

**Figure 1:**
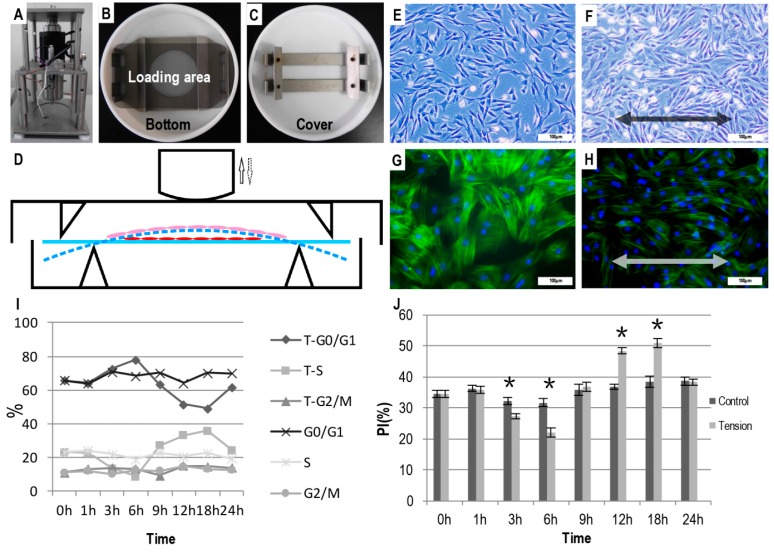
Illustration of the four-point bending apparatus and tensile stress on the morphology and proliferation of OCCM-30 cells (immortalized osteocalcin positive cementoblasts). (**A**) The Forcel^®^ four-point bending apparatus; (**B**) the stretching dish and (**C**) the force-loading plate; (**D**) Schematic graph depicting the administration of cyclic uniaxial tensile stress on the OCCM-30 cells. Light (**E**,**F**) or fluorescent (**G**,**H**) micrographs depicting the morphology and actin alignment of the cells with (**F**,**H**) or without cyclic tensile stress (**E**,**G**). The arrows in (**D**) indicated the direction of the cyclic pressure. The blue solid line and the blue dash line in (**D**) indicated the original and bended forms of cell culture plates under the cyclic tensile forces. The red and pink oblates indicated the cementoblasts that were upstretched or stretched under the cyclic tensile forces. Arrows in (**F**) and (**H**) indicated the direction of the cyclic tensile stress. Bar = 100 µm. Graphs depicting the results of flow cytometry analyses for the ratio of cell cycle phases (**I**) and the proliferation index (PI) (**J**) with or without tensile stress for certain durations. * *p* < 0.05.

After 24 h of tensile loading, the randomly-oriented cementoblasts had already reorganized with their axes parallel with the direction of the loading (Figure 1F). Consistently, the fluorescent staining also indicated that the cytoskeleton of the cementoblasts rearranged along the direction of the loading (Figure 1H). In contrast, the cells without loading remained randomly distributed (Figure 1E,G).

During the tensile stress loading, phase S and the PI of the cementoblasts showed an S-shaped trend: they significantly decreased at 3 and 6 h; the S and PI phases significantly increased at 12 and 18 h. In comparison, these two parameters remained unchanged for the cementoblasts without any loading. At 24 h, the PI decreased to a similar level to that of the control cells (Figure 1I,J).

### 2.2. Tensile Stress-Induced Osteogenic Differentiation of OCCM-30 Cells

**Figure 2:**
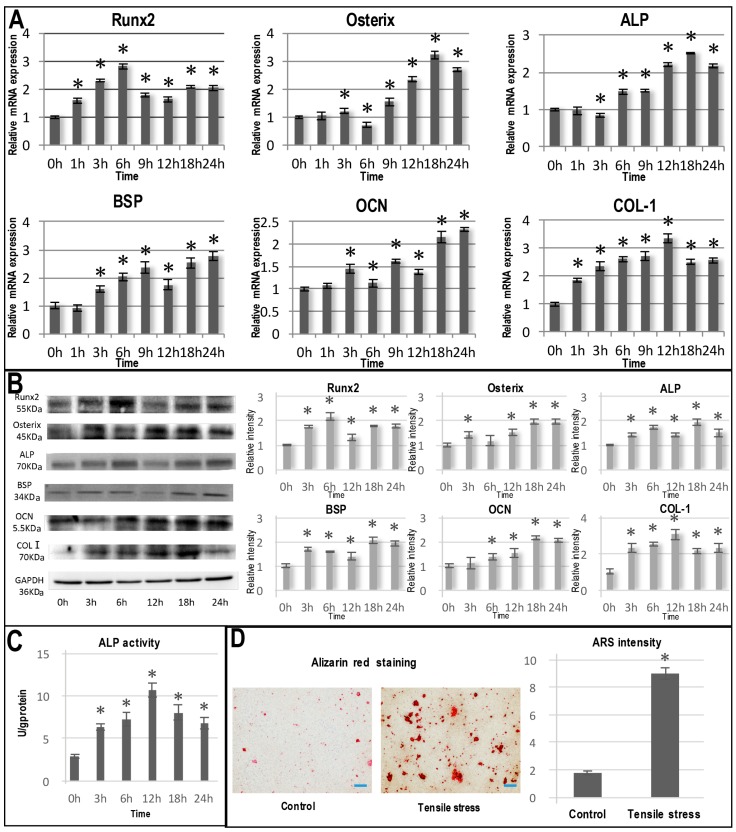
The cementoblastogenic differentiation of OCCM-30 cells under cyclic tensile stress stimulation. (**A**) Graphs depicting the relative mRNA expression of Runx2, Osterix, ALP, BSP, OCN and COL-I in OCCM 30 cells under tensile stress stimulation for 0, 1, 3, 6, 9, 12, 18 and 24 h; (**B**) Graphs depicting Western blot analyses for the protein expression of Runx2, Osterix, ALP, BSP, OCN and COL-I in OCCM 30 cells under tensile stress stimulation for 0, 3, 6, 12, 18 and 24 h; graphs depicting ALP activity (**C**) and Alizarin staining (**D**). Bar = 100 µm. * *p* < 0.05 in comparison with Time 0.

In comparison with other osteogenesis-related genes, Runx2 and COL-1 mRNA expression was significantly upregulated at as early as 1 h under the stimulation of tensile stress and increased dramatically with time (Figure 2A). The mRNA expressions of Runx2 and COL-1 reached peaks at 6 and 12 h, respectively. Thereafter, their mRNA expressions still maintained at an elevated level that was approximately two-times the level at Time 0. The significant upregulation of Osterix, ALP, BSP and OCN mRNA expression occurred after 3 h of mechanical stimulation and reached peaks at 18 or 24 h (Figure 2A). The mRNA expression patterns of these osteogenic genes were also reflected in the expressions of the corresponding proteins (Figure 2B).

Consistently, ALP activity was also significantly upregulated in the tensile stress group compared with the control (*p* < 0.05; Figure 2C). At seven days post-loading, Alizarin Red staining illustrated a significantly greater (4.68-fold) mineralization area in the loading group than the control (Figure 2D).

### 2.3. Identification of Differentially-Expressed miRNAs Caused by Cyclic Tensile Stress

**Figure 3:**
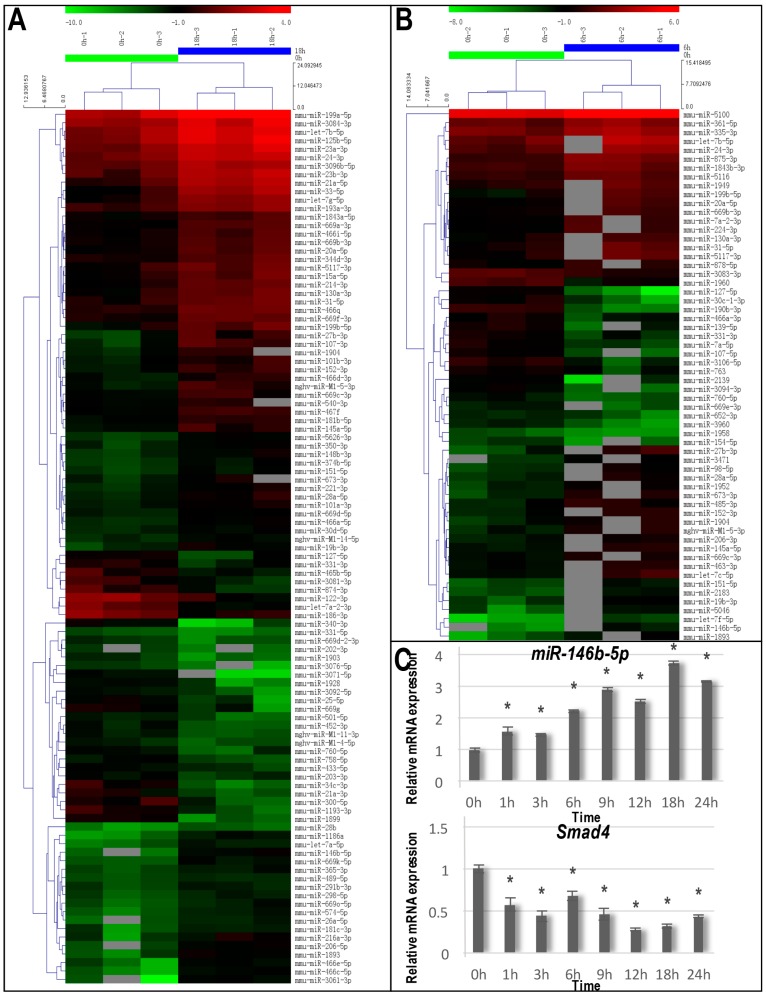
Identification of differentially-expressed micro-RNAs (miRNAs) stimulated by cyclic tensile stress and the qRT-PCR validation of miR-146b-5p and its target gene Smad4. (**A**) The hierarchical clustering of miRNAs differentially expressed after 18 h of tensile stress stimulation compared with the unstimulated cells; (**B**) The hierarchical clustering of miRNAs differentially expressed after 6 h of tensile stress stimulation compared with the unstimulated cells; (**C**) The graphs depicting the qRT-PCR analyses for the expression changes of miR-146b-5p and Smad4 mRNA under tensile stress stimulation for 0, 1, 3, 6, 9, 12, 18 and 24 h. * *p* < 0.05 in comparison with Time 0.

Compared with Time 0, the 6 and 18-h mechanical loadings resulted in significantly altered expression of 60 and 103 miRNAs, respectively. At 6 h, 40 miRNAs were upregulated (such as miR-146b-5p, miR-27b-3p and let-7f-5p) and 20 were downregulated (such as miR-127-5p, miR-3094-3p and miR-30c-1-3p) (Figure 3B; Table 1). At 18 h, 71 miRNAs were upregulated (such as miR-3061-3p, miR-466c-5p and miR-146b-5p), and 32 were downregulated (such as miR-3071-5p, miR-34c-3p and miR-1899) (Figure 3A). At each time point, the five most upregulated and downregulated miRNAs are listed in Table 1.

**Figure 4:**
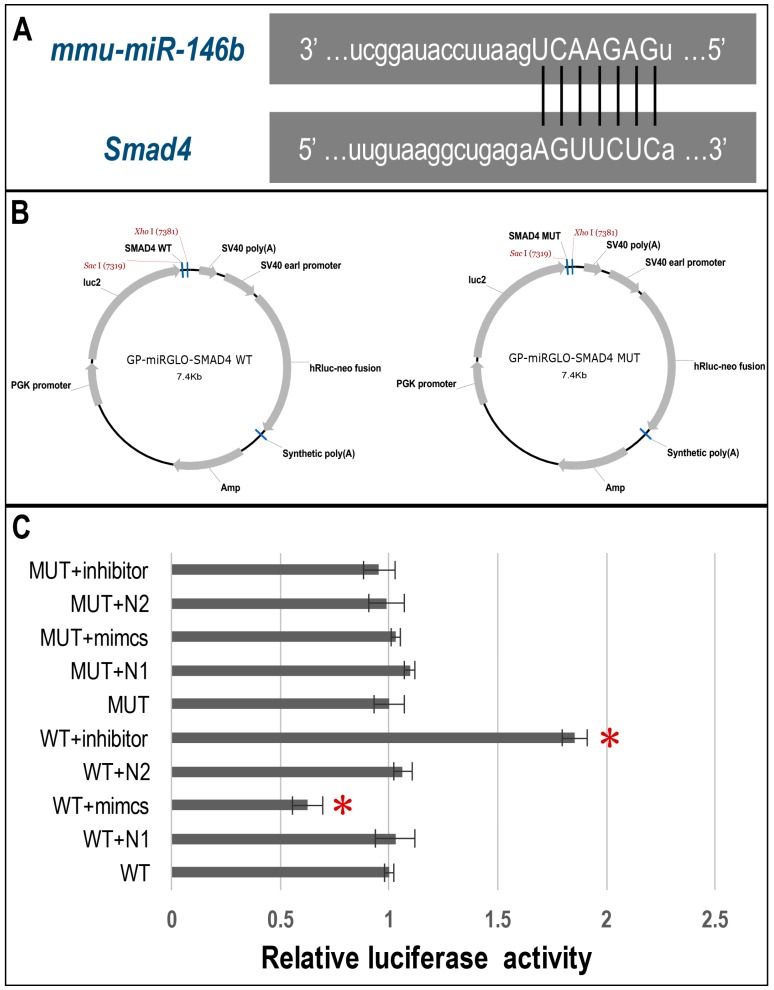
Dual-luciferase reporter assay of the direct binding of miR-146b-5p on the 3′ UTR of Smad4. (**A**) Bioinformatics analyses showed that miR-146b-5p was complementary to a sequence in the Smad4 3′ UTR; (**B**) The structure of pmirGLO-Smad4 3′ UTR-wild type (pmirGLO-Smad4-WT) and pmirGLO-Smad4 3′ UTR-mutation (pmirGLO-Smad4-MUT) luciferase reporter plasmids; (**C**) The pmirGLO-Smad4-WT and pmirGLO-Smad4-MUT reporter construct s were co-transfected with miR-146b-5p mimics, miR-146b-5p inhibitor and the negative control into 293 T cells. Co-transfection of pmirGLO-Smad4-WT with miR-146b-5p (WT + mimics) resulted in a significant reduction in the relative luciferase activity compared with the negative (WT + N1) or blank control (WT) (*p* < 0.05); furthermore, the co-transfection of pmirGLO-Smad4-WT with miR-146b-5p inhibitor (WT + inhibitor) resulted in a significant increase in relative luciferase activity compared with the negative (WT + N2) or blank control (WT) (*p* < 0.05). In contrast, the co-transfection of pmirGLO-Smad4-MUT with miR-146b-5p mimics or inhibitor showed no statistically-significant changes in relative luciferase activity (*p* > 0.05). * *p* < 0.05.

The pathway analysis demonstrated that after both 6 and 18 h of tensile stress, the mitogen-activated protein kinase MAPK signaling pathway, which is a pathway associated with cancer, the Wnt signaling pathway and the TGFβ/BMP-Smad signaling pathway were significantly related to the changes (*p* < 0.05; Table 2). Previous studies have suggested that both Wnt and TGFβ/BMP-Smad signaling pathways were highly involved in cementoblastogenesis. Consequently, we attempted to identify the related miRNAs and their potential targets in the categories of these two pathways. miRNA target prediction of screened miRNAs was carried out using bioinformatics analyses. We found that the Smad4 3′ UTR possessed a 7-nt seed match site for the screening-identified miR-146b-5p (Figure 4A). Based on these results, we hypothesized that Smad4 3′ UTR mRNA was a potential target gene of miR-146b-5p and selected miR-146b-5p as the focus of further investigation.

### 2.4. Validation of the miRNA and mRNA Expression

To further confirm the microarray results, we performed qRT-PCR to examine the time-dependent expression levels of miR-146b-5p and its predicted target gene Smad4 in the stretched group. The results showed that under cyclic tensile stress, miR-146b-5p significantly increased in a time-dependent manner and achieved a peak at 18 h compared to the unloaded control group. However, a significant decrease in the expression of Smad4 was confirmed in the tensile stress group with its lowest expression at 18 h (Figure 3C). Taken together, these results suggest that Smad4 would be the target gene of miR-146b-5p.

### 2.5. Dual-Luciferase Reporter Assay of Direct Binding of miR-146b-5p on the 3′ UTR of Smad4

Co-transfection of pmirGLO-Smad4-WT with miR-146b-5p (WT + mimics) in 293 T cells resulted in a significant reduction in the relative luciferase activity compared with the negative (WT + N1) or blank controls (WT) (*p* < 0.05; Figure 4C). Furthermore, the co-transfection of pmirGLO-Smad4-WT with miR-146b-5p inhibitor (WT + inhibitor) resulted in a significant increase in the relative luciferase activity compared with the negative (WT + N2) or blank controls (WT) (*p* < 0.05; Figure 4C). In contrast, co-transfection of pmirGLO-Smad4-MUT with miR-146b-5p mimics or inhibitor showed no statistically-significant changes in the relative luciferase activity (*p* > 0.05; Figure 4C).

## 3. Discussion

Cementoblasts, which are the major cells in cementum, play a critical role in sensing various biological (chemical or mechanical) signals and maintaining the integrity of cementum by undergoing the process of cementoblastogenesis. Tensile stress that is produced during masticatory activity is indispensable to facilitate moderate metabolic activity of cementoblasts, which thereby maintains the healthy anchorage of teeth [21]. The promoting effect of tensile stress on cementoblastogenesis can also be corroborated by the significantly thickened cementum during orthodontic tooth movement [22]. Hitherto, the extent to which post-translational modulating mechanisms, such as miRNA, are involved in the mechanical loading-induced cementoblastogenesis has been unknown. In this study, we, for the first time, screened the miRNA expression during tensile stress-induced cementoblastogenesis of mature cementoblast cells and validated their targets. Our data suggest a potential role of miR-146b-5p and its target Smad4 in this biological event.

In this study, we adopted the immortalized cementoblast cell line (OOCM-30), which is a well-established and widely-recognized mature cementoblast cell line [17,23,24,25,26]. The OCCM-30 cell line was first isolated from OC-TAG (the SV40 large T-antigen (TAg) was integrated into the osteocalcin (OC) promoter) transgenic mice in Martha Somerman’s lab at the University of Michigan [27]. These mice contained a construct that used the SV-40 T antigen under the direction of the osteocalcin promoter. In this situation, when cells were isolated from the tooth root surface of the OC-TAG mice, only the cells that expressed osteocalcin (i.e., cementoblasts) were capable of surviving serial passages in vitro. These cells exhibited the identical properties as the cementoblasts in vivo, for example, they expressed high levels of cementoblast markers, such as BSP and OCN, and could undergo mineralization in vitro [27]. This cell line provides a convenient in vitro model to study cementogenesis. In previous reports, OCCM-30 cells have been shown to express some dentin markers, such as dentin matrix protein 1 (DMP-1) [23], while other dentin markers, such as CEMP1, were not investigated. Further experiments should be performed to characterize the potential role of CEMP1 in cyclic tensile stress-stimulated cementoblastogenesis of OCCM-30 cells.

In recent years, in vitro cell loading systems have been widely used to investigate the stress-induced changes in diverse cells, i.e., osteoblast, adipose-derived stem cells (ASCs), bone marrow stromal cells (BMSCs) and PDLSCs [1,6,11,28,29]. Cementoblasts have also been reported to be mechanosensitive. Their morphology, proliferation and cementoblastogenesis could be significantly upregulated by tensile or compressive stress [5,6,11]. We selected 2000 µstrain of stress to simulate physiological stimulation, since it is widely considered equivalent to physiological loading in orthodontic tooth movement [5,11,30].

Consistent with previous findings and when treated with cyclic tensile stress, OCCM-30 cells that were randomly oriented (Figure 1E,G) underwent rearrangement with their axes parallel aligning with the direction of the tensile stress (Figure 1F,H). This rearrangement process might account for the inhibited proliferation 3–6 h post-treatment, which was demonstrated by the decreased ratio of the S phase, the increased ratio of the G0/G1 (Gap 0 (resting) phase/Gap1 phase in cell cycle) phase and the decreased PI compared to the untreated control (Figure 1I,J). Thereafter, the proliferation of the treated cementoblasts recovered 9 h post-treatment and significantly increased at 12 and 18 h (Figure 1J). Accordingly, at 12 and 18 h, the treated cementoblasts showed a significantly greater ratio of the S phase and a significantly lower ratio of the G0/G1 phase. In comparison, the PI of untreated cells remained stable during the 24-h monitoring span.

Apart from cell proliferation, tensile stress could also significantly promote cementoblastogenic activities. Among all of the critical genes for cementoblastogenesis, Runx2 and collagen I mRNA were significantly upregulated at as early as 1 h post-treatment (Figure 2A). Both genes showed an initial time-dependent increasing pattern after which their expression decreased, but remained significantly greater than at Time 0. From 3–9 h, other cementoblastogenesis-related genes, such as Osterix and BSP, also steadily increased and maintained a significantly greater level than at Time 0 (Figure 2A). The promoting effect of mechanical tensile stress was further confirmed by the corresponding protein levels (Figure 2B). The results of the ALP activity assay, the ALP staining and the Alizarin Red staining also corroborated that the tensile stress could promote the osteogenic differentiation and subsequent mineralization of OCCM-30 cells (Figure 2C,D). These results were in accordance with Huang et al. and Yu et al. who found an increase in the expression of OPN and BSP after 2000 μstrain tensile stimulation in OCCM-30 cells, and the elevation of these makers was greater under the application of 2000 µstrain than 4000 µstrain [6,11]. In fact, the promotion towards an osteoblastogenesis-like phenotype is a common biological effect of tensile stress for various periodontal cells, such as PDLSCs and osteoblasts. For example, tensile stress could up-regulate a series of osteoblastogenesis-like phenotype genes, such as BMP2, BMP6, ALP, SOX9, MSX1 and VEGFA [31]. Shen et al. also discovered that both the mRNA and proteins of Runx2, ALP and OCN in PDLSCs were significantly upregulated after the mechanical loading for 6, 12 and 24 h compared to levels in untreated PDLSCs [28]. These findings present a biological mechanism accounting for the promoting effect of masticatory forces on the anchorage and integrity of periodontal cementum.

Until now, the molecular mechanisms accounting for the effect of tensile stress on osteoblastogenesis-like phenotype changes remained to be clarified. One potential mediator is the classical molecules for mechanosensitivity, such as PGE2 and COX-2 [32]. Rego et al. showed that tensile stress could significantly upregulate the COX-2 mRNA expression after 1 h and PGE2 production after 6 h of treatment [1]. Furthermore, tensile stress could also significantly enhance the mRNA expression of osteoblastogenesis-like phenotypic genes, such as BMP-2 and OCN. The promoting effect of tensile stress on BMP-2 gene expression is mediated at the transcriptional level, but not at the post-transcriptional level [33]. Furthermore, the upregulation of BMP-2 was, at least partially, mediated by the ERK and p38 MAPK pathways. As a mechanosensitive molecule, COX-2 gene expression is observed to be dependent on the ERK1/2 and p38 MAP kinase pathways and induces prostaglandin E2 (PGE2) biosynthesis. The inhibition of the endogenous production of PGE2 by NS-398, which is a selective COX-2 inhibitor, could completely abolish the up-regulation of ALP and BMP-2 mRNA expression induced by tensile stress [1,33]. Such inhibition by NS-398 could be completely restored through the addition of exogenous PGE2 [1]. These findings indicate the indispensable role of the ERK/p38-COX-2-PGE2 pathway in the tensile stress-induced cementoblastogenesis and endogenous production of BMP-2 [1,33]. However, in these studies, evidence was provided to prove the relation between endogenous BMP-2 and the cementoblastogenesis. Boabaid et al. suggested an involvement of the MAPK pathway in cementoblast behavior, since the addition of the MAPK inhibitor suppressed OPN expression in LRAP-treated cementoblasts [34]. Apart from MAPK signaling, Wnt/β-catenin may also significantly influence cementoblastogenesis. Tensile stress could significantly enhance β-catenin production and Runx2 mRNA expression. Furthermore, the upregulation of Runx2 mRNA expression by tensile stress was completely inhibited when β-catenin production was suppressed [35]. However, the effect of Wnt/β-catenin signaling was still ambiguous. Osterix acted as an important molecule to promote osteoblastogenesis-like differentiation in periodontal ligament cells under tensile stress stimulation [36]. The Osterix-positive cells were mainly distributed on the surfaces of alveolar bone and cementum. The overexpressed Osterix could promote cementoblast differentiation by maintaining a low level of Wnt-β-catenin through the direct upregulation of Dickkopf-related protein 1 [37]. One possible explanation for these controversial findings might be that the canonical Wnt signaling might differently influence cementoblastogenesis under the presence and absence of tensile stress. In fact, the role of BMP-Smad signaling, which is a well-established osteogenic differentiation pathway, was also ambiguous in the process of the cementoblastogenesis of mature cementoblasts. Exogenous BMP-2 could promote cementoblastic differentiation of dental follicle cells [15,16] and PDL mesenchymal stem cells [38] and at 0–300 ng/mL could dose dependently inhibit the mineralization of cementoblasts [17]. Conversely, exogenous BMP-7 could strongly upregulate cementoblastogenesis of mature cementoblast cells [39]. Furthermore, TGF-β signaling plays a major role as one of the upstream regulators of Osterix in cementoblast differentiation and cementum formation [25]. All of these data suggest that cementoblastogenesis is modulated by TGF-β/BMP in a more complicated manner than osteoblastogenesis.

In this study, we attempted to explore whether post-translational modulation mechanisms were involved in tensile stress-induced cementoblastogenesis. For this purpose, we applied cyclic tensile stress to a mature cementoblast cell line and screened the miRNA expression using a microarray assay. Our data show that the expression of 60 and 103 miRNAs was significantly altered after 6 and 18 h (Figure 3A,B and Table 1) compared with unstimulated cells (fold change > 2, *p* < 0.05). Since no study has reported miRNA expression and the targets in cementoblasts, we performed a bioinformatics analysis by integrating a pathway analysis of potential miRNA targets with our miRNA expression data. With this method, we selected six potential signaling pathways, such as the TGFβ/BMP-Smad signaling pathway, the Wnt signaling pathway, the MAPK signaling pathway and the T cell receptor signaling pathway (Table 2). Since previous studies have suggested that both the Wnt and TGFβ/BMP-Smad signaling pathways were highly involved in cementoblastogenesis, we attempted to focus the related miRNAs and their potential targets in the categories of these two pathways. We performed qRT-PCR to further identify the expression of miRNA and their potential target genes. Many miRNA target genes were excluded because there were unmatchable expression trends of miRNA and the target gene, there were no binding site of the miRNA to target genes or there were inconsistent PCR and microarray analysis results. In this process, we found that miR-146b-5p gradually increased in OCCM-30 cells under tensile stress compared with the control. Correspondingly, the qRT-PCR result showed that the Smad4 gene expression decreased at the corresponding time points (Figure 3C). Based on the target prediction programs, we found the 3′ UTR of Smad4 possesses a 7-nt match site to the miR-146b-5p seed region (Figure 4A). Furthermore, we confirmed that the Smad4 3′ UTR was the direct target of miR-146b-5p using a dual-luciferase reporter assay (Figure 4C).

In recent years, many studies have shown the expression of miR-146b-5p in the development and progression of different tumors, such as papillary thyroid carcinoma [40,41], glioma [42], pancreatic cancer [43] and osteosarcoma [44]. In thyroid cancer, miR-146b-5p was highly expressed and inhibited Smad4 gene expression, which favored the resistance of tumors to a TGF-β inhibitory signal [41]. Our study is the first study to suggest a potential role of miR-146b-5p/Smad4 in tensile stress-induced cementoblastogenesis. Smad4 is a common mediator of both BMP and TGF-β signaling. Xu et al. [44] found that miR-146b-5p was highly expressed in human osteosarcoma tissues, and it contributing to chemoresistance of osteosarcoma. Furthermore, miR-146b-5p overexpression has been shown to promote migration and invasiveness through the downregulation of ZNRF3 through the modulation of the Wnt/β-catenin signaling pathway [44]. In our experiments, our validation process did not show a direct target of miR-146b-5p to the Wnt/β-catenin signaling pathway. Instead, we showed that miR-146b-5p could be significantly upregulated during tensile stress-induced cementoblastogenesis and caused the expression of the *Smad4* gene. The exact role of the miR-146b-5p/Smad4 post-translational modulation mechanisms in the tensile stress-induced cementoblastogenesis requires clarification. Based on the findings of Zhao et al. where BMP-2 inhibited cementoblastogenesis [17], one possible role is that the tensile stress could enhance cementoblastogenesis by suppressing the inhibitory effect of BMP-2 by enhancing miR-146b-5p and, thus, reducing Smad4. When considering the promoting effect of BMP-7 and TGF-β on cementoblastogenesis, the miR-146b-5p/Smad4 axis could also be a negative feedback to tensile stress-induced cementoblastogenesis. In fact, hitherto, there has been no study to directly prove the effects of endogenous BMPs/TGF-β in tensile stress-induced cementoblastogenesis. Further studies should be performed to clarify the molecular mechanisms. In addition, the Runx2 gene was upregulated at as early as 1 h in this study, which suggested a rapid molecular mechanism, such as the activation of cytoplasmic kinases and transcription factors.

In recent years, increasing data have demonstrated that the signaling network in skeletal development and new bone formation is highly time-dependent and space-specific. Both positive, negative and synergistic effects could be observed when TGF-β/BMP interacts with the pathways of Akt/mTOR, Wnt, MAPK, Notch, Hedgehog (Hh) to regulate the effects of TGF-β/BMP-induced signaling in bone remodeling [45]. These results were in accordance with the pathway analysis in this study; when tensile stress was applied for 6 and 18 h, the MAPK signaling pathway, which is a pathway associated with cancer, Wnt signaling pathway and TGFβ/BMP-Smad signaling pathway were significantly changed (Table 2). Similarly, the contrasting roles could be attributed to the different targets of miR-146b-5p in different cell types. miR-146b-5p may be involved in a complex network of gene expression regulation that could tissue- and stage-dependently target different mRNA species in each circumstance [41].

One limitation of this study is the final identification of the role of the miR-146b-5p/Smad4 axis in tensile stress-induced cementoblastogenesis. Further studies should be performed to identify not only this aspect, but also the molecular link between tensile stress and the miR-146b-5p/Smad4 axis. Another limitation was that we mainly focused on TGFβ/BMP and the Wnt signaling pathway. Further experiments should be performed to assess other signaling pathways, such MAPK, which is suggested to play an important role in cementoblastogenesis [34]. Caution should be taken to extrapolate the current results to predict the miRNA profiling in other periodontal cell types, since this mature cementoblast cell line showed different phenotypes from osteoblasts or PDLSCs.

## 4. Materials and Methods

### 4.1. Cell Culture

Immortalized murine cementoblast-like cells (OCCM-30) were cultured in Dulbecco′s Modified Eagle′s Medium (DMEM; Gibco, St. Louis, MO, USA) supplemented with 10% fetal bovine serum (FBS, Gibco, CA, USA), 100 U/mL penicillin and 100 µg/mL streptomycin at 37 °C in a humidified atmosphere of 5% CO_2_/95% air. The medium was changed every 2 days. Once 80% confluence was achieved, cells were trypsinized for the subsequent experiments. Three samples per group per time point were used in the following experiments.

### 4.2. Application of Cyclic Tensile Force

Mechanical stimulation was administered using a four-point bending apparatus as reported previously (Figure 1A) [5,6,30]. The force-loading plates were made from the bottom of a 75-cm^2^ cell culture flask (Corning, NY, USA) and were 8.5 cm × 4.0 cm in size and 1.5 mm thick. The sharp corner was removed to avoid stress accumulation (Figure 1B). OCCM-30 cells were seeded onto these plates at a density of 4 × 10^5^ cells/cm^2^. After the cultures had reached 80% confluence, the cells were serum deprived for 24 h with serum-free medium. Subsequently, the force-loading plates were placed in stretching dishes (Figure 1B–D) that were filled with medium and subjected to a cyclic uniaxial tensile stress (0.5 Hz, 2000 µstrain) for different time periods, and the control groups were cultured without stretching.

### 4.3. Cell Morphology and Cell Proliferation Assay

Twenty-four hours after cyclic tensile stress stimulation, the cell morphology was observed using an inverted microscope (Olympus, Tokyo, Japan) with a cell imaging system. Thereafter, the cells were fixed with 4% paraformaldehyde (PFA; Sigma, St. Louis, MO, USA) at room temperature for 15 min and permeabilized with 0.1% Triton X-100 in phosphate-buffered saline (PBS) at room temperature for 5 min. After rinsing with PBS twice, the cells were stained with a 5-µg/mL fluorescein isothiocyanate (FITC)-phalloidin working solution (Sigma) by incubating the cells at 37 °C in the dark for 60 min. After thoroughly washing with PBS, the cells were subsequently counter-stained with Hoechst stain (Sigma) by incubation at 37 °C in the dark for another 10 min. Finally, florescence microscopy (Olympus, Tokyo, Japan) was used to visualize the cytoskeleton of the cells.

The cell cycle and proliferation index (PI) were evaluated using flow cytometry (FCM; Beckman Coulter, Brea, CA, USA). At 0, 1 3, 6, 9, 12, 18 and 24 h after the administration of the mechanical forces, the cells were trypsinized, harvested and stored in 70% cold ethanol overnight at 4 °C. Thereafter, the fixed cells were centrifuged and washed twice with PBS and stained with 400 µL of a propidium iodide solution (10 µg/mL) (Sigma) at room temperature for 30 min. Subsequently, samples were analyzed in terms of DNA content; the various cell cycles were evaluated; and the PI was calculated separately. Samples in the control group were collected at the corresponding time points, and each group was carried out in triplicate.

### 4.4. Real-Time PCR Analysis

After the tensile stress was applied for 0, 1, 3, 6, 9, 12, 18 and 24 h, the cells were washed twice with PBS. We extracted the total RNA using TRIzol (Invitrogen, Carlsbad, CA, USA) according to the manufacturer′s protocol. Thereafter, we quantified the total RNA with a Nanodrop spectrophotometer (Thermo, Waltham, MA, USA) at an absorbance (A) of 260 nm. Two micrograms of total RNA per sample were then subjected to reverse transcription using the Takara PrimeScript^®^ RT reagent kit (TaKaRa, Otsu, Japan) according to the manufacturer′s protocol. Each real-time PCR was carried out in triplicate with a total of 20 µL of reaction mixture using Takara SYBR^®^ Premix Ex Taq™ II (TaKaRa) in an ABI PRISM 7300 real-time PCR system. Primers used for the real-time PCR analysis are presented in Table 3. Relative expression levels were calculated using the 2^−ΔΔ*C*t^ method. The expression of specific mRNA from the unloaded cells was used as the baseline, and GAPDH was used as an endogenous control.

### 4.5. Western Blotting

After the tensile stress was administered for 0, 3, 6, 12, 18 and 24 h, the cells were washed twice with PBS and then harvested using TRIzol (Invitrogen) according to the manufacturer′s protocol. The protein concentration was measured using a BCA protein assay reagent kit (Beyotime, Shanghai, China). Equal aliquots of protein samples were separated using SDS-PAGE and then electrotransferred onto polyvinylidene fluoride membranes (PVDF; Millipore, Billerica, MA, USA). The membranes were then blocked with 5% lipid-free milk in Tris-buffered saline with 0.1% Tween (TBST) for 2 h at 37 °C, which was followed by an incubation with primary antibodies at 4 °C overnight. The membrane was washed with TBST 6 times for 5 min and was exposed to HRP-conjugated goat anti-rabbit secondary antibodies (Santa Cruz, Santa Cruz, CA, USA) for 2 h at 37 °C. Immunoreactive proteins were visualized using a chemiluminescence kit (Millipore). Band intensities were determined using the ChemiDoc XRS Gel documentation system and the Quantity One software (Bio-Rad, Hercules, CA, USA). Loading differences were normalized by assessing the housekeeping protein (GAPDH) in each sample. All primary antibodies, including anti-Runx2, anti-Osterix, anti-BSP, anti-OCN, anti-ALP, anti-COL-I and anti-GAPDH, were purchased from Abcam (Abcam, Cambridge, MA, USA).

### 4.6. Alkaline Phosphatase and In Vitro Mineralization Assay

To evaluate the effect of tensile stress on mineralization, the cells were cultured in osteogenic medium containing 50 µg/mL ascorbic acid, 10 mM β-glycerophosphate and 10^−8^ M dexamethasone. After that, the cyclic tensile stress was applied for 24 h, the cells were washed with PBS, fixed with 4% PFA and stained with alkaline phosphatase substrate according to the manufacturer′s protocol (Abcam, Hangzhou, China) to verify early mineralization activity. The cells cultured in the same condition, but without the tensile stress were used as controls.

ALP activity was measured with an ALP activity kit according to the manufacturer′s protocol (Jiancheng, Nanjing, China). The activity was normalized using a BCA protein assay reagent kit (Beyotime, Shanghai, China) with triplicates per group.

Subsequently, cyclic tensile stress was applied for 24 h, and the cells were cultured in osteogenic media for 7 days. Thereafter, the cultured cells were fixed with cold methanol and stained with a 10% alizarin red solution (Sigma). The mineralized nodules were identified as red spots on the culture dish.

### 4.7. miRNA Microarray and Bioinformatic Analysis

Hitherto, there have been no reports to show the modulating effect of cyclic tensile stress on miRNA in OCCM-30 cementoblasts. We found that most of the cementoblastogenic markers reached a peak 6 or 18 h post-stimulation. Consequently, we performed a microarray to screen the miRNA profile at 0, 6 and 18 h after the administration of tensile stress. At each time point, the total RNA was extracted using TRIzol (Invitrogen) and miRNeasy mini kits (QIAGEN, Hilden, Germany) according to the manufacturers′ protocols. RNA quality and quantity were assessed using a Nanodrop spectrophotometer (Thermo). We labelled the samples using the miRCURY Array Labeling kit (Exiqon) and hybridized with the miRCURYTM LNA Array (v.18.0; Exiqon). After hybridization, we harvested the slides, washed 6 times using a wash buffer kit (Exiqon, Vedbaek, Denmark) and dried with centrifugation for 5 min at 1000 rpm. Finally, we scanned the slides using the Axon GenePix 4000B microarray scanner (Axon Instruments, Sunnyvale, CA, USA). Thereby obtained images were then imported into GenePix Pro 6.0 software (Axon Instruments) for grid alignment and data extraction.

After the data were acquired, miRNA target prediction was carried out using the following databases: TargetScan (Available online: http://www.targetscan.org), microRNA.org (Available online: http://www.microrna.org), Pictar (Available online: http://pictar.mdc-berlin.de) and DIANA MicroT (Available online: http://diana.cslab.ece.ntua.gr/microT/).

### 4.8. Validation of Microarray Results by Quantitative Real-Time PCR

According to the microarray and bioinformatic analysis, the selected miRNA of miR-146b-5p and its predicted target gene Smad4 were used for real-time reverse transcription polymerase chain reaction (RT-PCR) validation using the method described in Section 4.4. The primers used for the real-time PCR analysis are presented in Table 3.

### 4.9. Dual Luciferase Reporter Assay

A luciferase assay was performed to validate that Smad4 was a target gene of miR-146b-5p in tensile stress-stimulated OCCM-30 cells. A synthetic fragment of the Smad4 3′ UTR containing the putative binding site for miR-146b-5p or the mutant site was inserted between the XhoI and Sac I cleavage sites of the pmirGLO reporter vector (Promega, Madison, WI, USA) downstream of the firefly luciferase reporter gene (luc2) to construct the recombinant reporter vectors pmirGLO-Smad4 3′ UTR-wild type (pmirGLO-Smad4-WT) and pmirGLO-Smad4 3′ UTR-mutation (pmirGLO-Smad4-MUT) (Figure 4B). The sequences for Smad4 3′ UTR-WT and Smad4 3′ UTR-MUT are listed in Table 4. The mimic was synthesized according to the sequence of miR-146b-5p. A Smad4 3′ UTR segment containing the predicted miR-146b-5p binding site was cloned into the pGL3-control vector (Promega) downstream of the firefly luciferase gene after which the 3′ UTR luciferase reporter was obtained. The miR-146b-5p mimic inhibitor used in the present study was synthesized and provided by GenePharm (Table 4). For luciferase reporter assays, 293 T cells were seeded into 96-well plates at 70-80% confluence 24 h before transfection. Cells were then co-transfected with corresponding reporter constructs. We determined the Firefly and Renilla luciferase activities 48 h post transfection using the Dual Luciferase Reporter Gene Assay Kit (Beyotime). We normalized the firefly values to Renilla luciferase.

### 4.10. Statistics

The data are presented as the mean ± standard error (SE) for each group and were analyzed using SPSS software (Version 22, SPSS, IBM, Armonk, NY, USA). The non-parametric Mann–Whitney test was used for comparisons between each time points with Time 0 within the same group, between 2 groups at the same time point and between two selected groups. Differences were considered significant when *p* < 0.05.

## 5. Conclusions

In this study, we screened the miRNA profile during the cyclic tensile stress-induced cementoblasts. After bioinformatics analysis, we confirmed the concomitant upregulation of miR-146b-5p and downregulation of Smad4 during this biological event. The direct binding of miR-146b-5p to the three prime untranslated region (3′ UTR) of Smad4 was established using a dual-luciferase reporter assay. Taken together, these results suggested an important involvement of miR-146b-5p and its target gene Smad4 in the cementoblastogenesis of mature cementoblasts.

## Figures and Tables

**Table 1:** The 5 most upregulated and downregulated miRNAs after 6 or 18 h of tensile stress stimulation in comparison with Time 0.

Name	Fold Change	*p*-Value	Up/Down
6 vs. 0 h			
mmu-miR-146b-5p	8.149	0.0423	Up
mmu-miR-27b-3p	7.918	0.0246	Up
mmu-let-7f-5p	7.669	0.0047	Up
mmu-miR-1893	5.628	0.0014	Up
mmu-miR-673-3p	4.688	0.0014	Up
mmu-miR-127-5p	0.057	0.0289	Down
mmu-miR-3094-3p	0.139	0.0331	Down
mmu-miR-30c-1-3p	0.149	0.0227	Down
mmu-miR-107-5p	0.150	0.0108	Down
mmu-miR-3106-5p	0.205	0.0048	Down
18 vs. 0 h			
mmu-miR-3061-3p	11.470	0.0107	Up
mmu-miR-466c-5p	11.465	0.0020	Up
mmu-miR-146b-5p	11.257	0.0469	Up
mmu-miR-1186a	10.843	0.0065	Up
mmu-miR-1893	7.214	0.0052	Up
mmu-miR-3071-5p	0.009	0.0101	Down
mmu-miR-34c-3p	0.058	0.01162	Down
mmu-miR-1899	0.066	0.03673	Down
mmu-miR-340-3p	0.091	0.0293	Down
mmu-miR-21a-3p	0.123	0.0373	Down

**Table 2:** Pathway analyses of differentially-expressed miRNA-related signaling pathways and example genes after 6 and 18 h of tensile stress stimulation.

Signaling Pathway	Example Genes
6 vs. 0 h	
MAPK signaling pathway	*MAPK1*, *EGFR*, *FGF1*, *FGF10*, *TGFβ2*, *TGFβR1*
T cell receptor signaling pathway	*MAPK1*, *KRAS*, *NRAS*, *IL5*, *IL2*, *IL10*, *SOS1*, *SOS2*
Chemokine signaling pathway	*MAPK1*, *KRAS*, *NRAS*, *SOS1*, *GRB2*, *PTK2*, *PIK3R1*
Pathways in cancer	*SMAD4*, *RUNX1*, *TGFβ2*, *TGFβR1*, *IGF1R*, *IGF1*, *BMP2*
Wnt signaling pathway	*DKK2*, *TCF3*, *APC*, *DKK3*, *RUNX2*
TGFβ/BMP-Smad signaling pathway	*SMAD4*, *TGFβ2*, *TGFβR1*, *BMP2*, *BMP3*
18 vs. 0 h	
MAPK signaling pathway	*FGFR1*, *FGFR2*, *FGF13*, *FGF5*, *TGFβR2*, *MAP3K1*
Pathways in cancer	*FGFR1*, *FGFR2*, *FGF5*, *SMAD4*, *TGFβR2*, *IGF1*, *RUNX2*
Cytokine-cytokine receptor interaction	*PDGFRβ*, *TGFBβ2*, *IL1*, *IL12β*, *IL6*
Wnt signaling pathway	*RHOA*, *APC*, *RUNX2*, *SMAD4*, *FZD4*, *FZD6*, *LRP6*, *DKK2*
TGFβ/BMP-Smad signaling pathway	*SMAD4*, *TGFβ2*, *TGFβR1*, *BMP2*, *BMP3*

**Table 3:** Constructed sequences and primers used in this study.

Gene	Primers (5′–3′)	GenBank Acc. No.	Length (bp)
*Runx2*	Forward-TTCAACGATCTGAGATTTGTGGG	NM_001145920	221
	Reverse-GGATGAGGAATGCGCCCTA		
*Osterix*	Forward-ATGGCGTCCTCTCTGCTTG	NM_130458	156
	Reverse-TGAAAGGTCAGCGTATGGCTT		
*ALP*	Forward-GATGTGGAATACGAACTGGATG	NM_007431	104
	Reverse-TGGGAATGCTTGTGTCTGG		
*BSP*	Forward-AGAGCGGTGAGTCTAAGGAGT	NM_001033418	90
	Reverse-TGCCCTTTCCGTTGTTGTCC		
*OCN*	Forward-ATCTTTCTGCTCACTCTGCTG	NM_001037939	117
	Reverse-CTTATTGCCCTCCTGCTTGG		
*COL-I*	Forward-CTGGCGGTTCAGGTCCAAT	NM_007742	141
	Reverse-TTCCAGGCAATCCACGAGC		
*GAPDH*	Forward-TGGTGAAGGTCGGTGTGAAC	NM_008084	231
	Reverse-GCTCCTGGAAGATGGTGATGG		
*SMAD4*	Forward-AGGTGGCCTGATCTACACAAG	NM_008540	110
	Reverse-ACCCGCTCATAGTGATATGGATT		

**Table 4:** The sequences for the vector constructs of SMAD4 3′ UTR-WT, SMAD4 3′ UTR-MUT, Mmu-miR-146b inhibitor and Mmu-miR-146b mimic. The mutation parts in the sequences of SMAD4 3′ UTR-MUT are highlighted with double underlines.

Vector Construct	Sequences
SMAD4 3′ UTR-WT	F-CCATGCCGAGGAGAGTCAGAGCTGCTGATTGTAAGGCTGAGAAGTTCTCACAGTTAAGCCAC
	R-TCGAGTGGCTTAACTGTGAGAACTTCTCAGCCTTACAATCAGCAGCTCTGACTCTCCTCGGCATGGAGCT
SMAD4 3′ UTR-MUT	F-CCATGCCGAGGAGAGTCAGAGCTGCTGATTGTAAGGCTGAGATCAAGAGTCAGTTAAGCCAC
	R-TCGAGTGGCTTAACTGACTCTTGATCTCAGCCTTACAATCAGCAGCTCTGACTCTCCTCGGCATGGAGCT
Mmu-miR-146b inhibitor	F-CAGCCTATGGAATTCAGTTCTCAACCGGTAGCCTATGGAATTCAGTTCTCAC
	R-TCGAGTGAGAACTGAATTCCATAGGCTACCGGTTGAGAACTGAATTCCATAGGCTGAGCT
Mmu-miR-146b mimics	F-CTGAGAACTGAATTCCATAGGCTACCGGTTGAGAACTGAATTCCATAGGCTC
	R-TCGAGAGCCTATGGAATTCAGTTCTCAACCGGTAGCCTATGGAATTCAGTTCTCAGAGCT
